# Racial/ethnic differences in the association between alcohol use and mortality among men living with HIV

**DOI:** 10.1186/s13722-017-0103-z

**Published:** 2018-01-22

**Authors:** Kara M. Bensley, Kathleen A. McGinnis, David A. Fiellin, Adam J. Gordon, Kevin L. Kraemer, Kendall J. Bryant, E. Jennifer Edelman, Stephen Crystal, Julie R. Gaither, P. Todd Korthuis, Brandon D. L. Marshall, India J. Ornelas, K. C. Gary Chan, Julia C. Dombrowski, John C. Fortney, Amy C. Justice, Emily C. Williams

**Affiliations:** 1 Department of Health Services, Magnuson Health Sciences Center, University of Washington School of Public Health, 1959 NE Pacific St, Box 357660, Seattle, WA 98195-7660 USA; 2 VA Health Services Research and Development (HSRD) Denver Seattle Center of Innovation for Veteran-Centered and Value-Driven Care, VA Puget Sound Healthcare System, 1660 S. Columbian Way, Mailstop S-152, Seattle, WA 98108 USA; 3 VA Connecticut Healthcare System, West Haven Campus, 950 Campbell Avenue, West Haven, CT 06516 USA; 4 Yale School of Medicine, 333 Cedar St, New Haven, CT 06510 USA; 5 Salt Lake City VA, 500 Foothill Dr, Salt Lake City, UT 84148 USA; 6 University of Utah, 201 Presidents Cir, Salt Lake City, UT 84112 USA; 7 VA Pittsburgh Healthcare System, University Drive, Pittsburgh, PA 15240 USA; 8 University of Pittsburgh School of Medicine, 3550 Terrace St, Pittsburgh, PA 15213 USA; 9 National Institute on Alcohol Abuse and Alcoholism, 5635 Fishers Ln, Rockville, MD 20852 USA; 10 Rutgers University, 7 College Ave, New Brunswick, NJ 08901 USA; 11 Oregon Health & Science University, 3181 SW Sam Jackson Park Rd, Portland, OR 97239 USA; 12 Brown University School of Public Health, 121 S Main St, Providence, RI 02903 USA; 13 University of Washington School of Medicine, 1959 NE Pacific St, Seattle, WA 98195 USA

**Keywords:** HIV, Alcohol use, Mortality risk, Racial/ethnic disparities, Veterans

## Abstract

**Background:**

Increasing alcohol use is associated with increased risk of mortality among patients living with HIV (PLWH). This association varies by race/ethnicity among general outpatients, but racial/ethnic variation has not been investigated among PLWH, among whom racial/ethnic minorities are disproportionately represented.

**Methods:**

VA electronic health record data from the Veterans Aging Cohort Study (2008–2012) were used to describe and compare mortality rates across race/ethnicity and levels of alcohol use defined by the Alcohol Use Disorders Identification Test-Consumption (AUDIT-C) questionnaire. Within each racial/ethnic group, Cox proportional hazards models, adjusted for age, disease severity, and comorbidities, compared mortality risk for moderate-risk (AUDIT-C = 4–7) and high-risk (AUDIT-C ≥ 8) relative to lower-risk (AUDIT-C = 1–3) alcohol use.

**Results:**

Mean follow-up time among black (n = 8518), Hispanic (n = 1353), and white (n = 7368) male PLWH with documented AUDIT-C screening (n = 17,239) was 4.3 years. Black PLWH had the highest mortality rate among patients reporting lower-risk alcohol use (2.9/100 person-years) relative to Hispanic and white PLWH (1.8 and 2.3, respectively) (*p* value for overall comparison = 0.011). Mortality risk was increased for patients reporting high-risk relative to lower-risk alcohol use in all racial/ethnic groups [black adjusted hazard ratio (AHR) = 1.36, 95% confidence interval (CI) 1.12–1.66; Hispanic AHR = 2.18, 95% CI 1.30–3.64; and white AHR = 2.04, 95% CI 1.61–2.58]. For only white PLWH, mortality risk was increased for patients reporting moderate-relative to lower-risk alcohol use (black AHR = 1.09, 95% CI 0.93–1.27; Hispanic AHR = 1.36, 95% CI 0.89–2.09; white AHR = 1.51, 95% CI 1.28–1.77).

**Conclusion:**

Among all PLWH, mortality risk was increased among patients reporting high-risk alcohol use across all racial/ethnic groups, but mortality risk was only increased among patients reporting moderate-risk relative to lower-risk alcohol use among white PLWH, and black patients appeared to have higher mortality risk relative to white patients at lower-risk levels of alcohol use. Findings of the present study further underscore the need to address unhealthy alcohol use among PLWH, and future research is needed to understand mechanisms underlying observed differences.

## Background

Alcohol use is common among people living with human immunodeficiency virus (HIV) and has known adverse effects on HIV-related outcomes. Alcohol use adversely influences health among patients living with HIV (PLWH) and is associated with lower receipt and poorer outcomes of HIV treatment, and ultimately with mortality among PLWH [1–5]. While no level of alcohol use appears to be “safe” for PLWH, risk of adverse outcomes is generally increased for those with higher severity of alcohol use relative to those who use alcohol at lower levels [1, 6].

HIV infection disproportionately impacts vulnerable populations [7] including racial/ethnic minorities. Mortality rates among PLWH also differ across racial/ethnic groups, with black PLWH having the highest mortality rates and Hispanic the lowest [8]. Racial/ethnic minority PLWH experience increased HIV stigma [9], socioeconomic disadvantage and increased persecution [10], and decreased access to care [3] that may make consequences of alcohol use worse for racial/ethnic minority [10] relative to white PLWH [11–15]. Due to these experiences, non-white PLWH may have increased risk of mortality associated with alcohol use relative to whites. Previous studies in non-HIV-specific populations demonstrate that racial/ethnic minorities have worse consequences at similar levels of alcohol use [16] and higher risk of mortality at lower levels of alcohol use than whites [17]. Thus, we hypothesize that racial/ethnic minority PLWH may also have worse consequences at similar levels of alcohol use and higher mortality risk at lower levels of alcohol use than white PLWH. Because risk factors and influences of health behaviors are likely to vary among racial/ethnic groups depending on their lived experiences, experts have called for studies to examine alcohol-related risk among vulnerable sub-populations of PLWH [5]. No prior study has examined whether the association between alcohol use and mortality varies across race/ethnicity among PLWH. Therefore, we evaluated whether the association between level of alcohol use and mortality differs across racial/ethnic groups in a national sample of PLWH.

## Methods

### Data source

The data source for this study is the Veterans Aging Cohort Study (VACS), an observational cohort of patients who receive care at the nationwide Veterans Health Administration (VA) starting October 1, 1997 [18]. VACS data were extracted from the VA Informatics and Computing Infrastructure data warehouse, a national VA repository that includes clinical and administrative data for all VA patients, as well as clinical alcohol screening data (collected using the Alcohol Use Disorders Identification Test Consumption (AUDIT-C) questionnaire among over 90% of VA outpatients) since 2008 [19].

### Sample

We included all male PLWH with a documented AUDIT-C alcohol screening between October 1, 2008 and March 30, 2012 who reported any alcohol use in the past year (AUDIT-C scores > 0) and were documented to be one or more of the three major racial/ethnic groups in the United States: black, Hispanic, and/or white. Women, other minority groups, and patients with AUDIT-C scores indicating patients who reported no alcohol use in the past year (AUDIT-C = 0) were excluded. Though research of this type is needed among women and other minority groups, associations between alcohol use and outcomes vary based on gender [20–22], and the numbers of both women and other minorities with HIV who also report alcohol use at high levels are very small in VACS. Exclusion of patients reporting no alcohol use was done because the measure of alcohol used (AUDIT-C score) does not differentiate between lifetime abstainers and those who have become abstinent due to other reasons (e.g., declining health) among patients reporting no alcohol use [1, 6].

### Predictor

Alcohol use was measured using the AUDIT-C, a validated screen for unhealthy alcohol use [23–25] and a measure of alcohol use severity [26, 27]. The AUDIT-C is also associated with phosphatidylethanol, a biomarker for alcohol use [28, 29]. AUDIT-C scores range from 0 to 12; scores of 0 represent patients reporting no alcohol use in the past year, and increasing scores are associated with increased risk of multiple medical outcomes [30, 31], alcohol use disorder symptoms [24–26, 32, 33], and mortality [4, 17, 28, 30, 34]. For this study, the first AUDIT-C documented during the study period for each patient was used to measure levels of alcohol use based on clinically meaningful AUDIT-C cut-off scores for male patients [25, 26]: lower-risk (AUDIT-C 1-3), moderate-risk (AUDIT-C 4-7), and high-risk (AUDIT-C 8-12). AUDIT-C screening is conducted as part of routine health screening at the VA, thus AUDIT-C scores are documented annually for over 90% of all established outpatients.

### Outcome

Time to death by any cause was specified as the primary outcome. Survival time was measured from date of the first AUDIT-C screening during the study period until death or loss to follow-up/end of study censoring, July 23, 2014 [35]. Death was measured using a validated measure created by combining four databases tracking deaths among VA patients: the Patient Treatment File, tracking hospital deaths within the VA System; the Beneficiary Identification Records Locating System, tracking VA death benefits; the Medicare Vital Status File; and the Social Security Death Master File, which has comparable completeness and accuracy to the National Death Index [36].

### Effect modifier

Race/ethnicity was categorized as non-Hispanic black, Hispanic, and non-Hispanic white [37]. Because patients can identify with multiple racial/ethnic groups, race/ethnicity was hierarchically coded, first as Hispanic ethnicity, and then as black and then white, consistent with single-race/ethnicity classification by rarest to most common racial/ethnic groups [38].

### Covariates

*Age* was categorized as < 50, 50–64, and 65 and older. Secondary measures of age were also derived: age as a continuous linear variable and age as a quadratic variable (age squared).

*HIV disease severity* Because HIV disease severity may confound the association between alcohol use and mortality [39], two measures of severity were described using the closest documented measure in the 6 months prior to and following AUDIT-C screening. These included: CD4 count (< 200, 200–500, > 500 cells/mm^3^) and a binary measure of viral load suppression (HIV-1 RNA < 200 copies/mL). In adjusted models, CD4 count was used to account for HIV disease severity as these measures are highly correlated. A binary measure of receipt of antiretroviral therapy (ART) at baseline AUDIT-C measure (at least one pharmacy fill prior to or within 7 days after baseline AUDIT-C measure) was described across racial ethnic groups. *Other comorbid conditions* were measured based on International Classification of Diseases 9th Edition (ICD-9 CM) codes documented any time between the beginning of VACS (10/1/97) and the baseline AUDIT-C measure. These included: non-AIDS related cancers (bladder, breast, buccal cavity and pharynx, digestive system, Hodgkin’s lymphoma, ill-defined cancer, kidney, leukemia, male genital system, melanomas, non-epithelial skin cancer, penile, prostate, stomach, testicular, ureter, urinary system), lung disease (chronic obstructive pulmonary disorder, asthma, or other lung disease), cardiovascular disease (congestive heart failure, myocardial infarction/coronary artery disease, peripheral vascular disease, ischemic stroke, stroke), hypertension, diabetes, hepatitis B, hepatitis C, anxiety, major depression, psychosis (e.g., schizophrenia), and post-traumatic stress disorder [40]. Body mass index (underweight < 18.5, normal/overweight = 18.6–29.9, obese > 30) was also included as HIV disease progression is associated with weight loss and wasting [41]; and higher BMI is associated with better HIV prognosis [42].

*Other substance use* measures included tobacco use (current, never, ever), determined based on documentation in an electronic clinical reminder [43], and current drug use (based on ICD-9 CM codes within 1 year prior to 6 months after AUDIT-C screening). These were also considered as covariates as both are associated with alcohol use and mortality.

### Analyses

Population characteristics, as well as levels of alcohol use and mortality rates, were described and compared across racial/ethnic groups; comparisons were done using Chi square tests of independence. To test racial/ethnic differences in the association between alcohol use and mortality, we fit a Cox Proportional Hazards model with time to death as the outcome and level of alcohol use as the predictor and tested a multiplicative interaction between racial/ethnic group and level of alcohol use. A Wald test was used to evaluate the overall significance of the interaction at p value < 0.05, and adjusted hazard ratios were calculated to estimate associations between level of alcohol use and mortality within each racial/ethnic group. Models were first unadjusted (Model 1) and then adjusted additionally for: age (Model 2), age and HIV disease severity based on CD4 count (Model 3), and age, HIV severity, receipt of ART, and comorbidities (Model 4). The iterative modeling strategy allowed for hypothesis-generation regarding what factors may be driving associations observed. Multiple imputation models were used to account for missing data in Cox Proportional Hazards models, particularly missing values for CD4 count, BMI, and smoking. To assess whether including age as a continuous linear or quadratic variable would better fit the model (i.e., address any residual confounding by age), models using categorical, linear, quadratic variables for age were tested among complete cases, and model fit was compared using Akaike Information Criterion. Tests of model fit indicated similar fits and results across models, thus age was used as a categorical variable in the primary analyses. All analyses were conducted in Stata Version 13 [44].

## Results

Eligible patients included 17,239 male PLWH; 49.4% were black, 7.9% were Hispanic, and 42.7% were white. The mean follow-up time was 4.3 years [Standard deviation (SD) = 1.3] for black, 4.3 (SD = 1.2) for Hispanic, and 4.4 (SD = 1.2) years for white PLWH.

Patient characteristics and levels of alcohol use differed across racial/ethnic groups (Table 1). Black patients had the highest prevalence of patients age 50–64, and were generally sicker than both white and Hispanic patients, having lower CD4 cell counts, higher viral loads, and higher proportions of current smoking, drug use, underweight or obese, hypertension, Hepatitis C, and PTSD. Black patients also included the highest proportion of patients reporting high and moderate risk alcohol use. Hispanic patients were generally younger, but had higher prevalence of hepatitis B than other racial/ethnic groups. White patients were generally older, and had better measures of HIV disease severity and receipt of ART than black patients (but similar to Hispanic patients). White PLWH also included the highest proportion with non-AIDS related cancer, lung disease, cardiovascular disease. White patients had the highest proportion of patients reporting lower-risk alcohol use.

**Table 1:** Patient characteristics and AUDIT-C categories across racial/ethnic groups and overall

	Black	Hispanic	White	p value	Overall
	n = 8518 (%)	n = 1353 (%)	n = 7368 (%)		n = 17, 239 (%)
*Demographic characteristics*					
Age					
< 50	38.2	39.5	37.7	< 0.001	38.1
50–64	55.9	52.3	49.1		52.7
65+	5.9	8.1	13.2		9.2
*HIV disease severity*					
CD4 count (< 200)^a^					
< 200	17.8	13.7	13.7	< 0.001	14.7
200–500	42.6	38.6	40.2		41.3
> 500	39.7	47.7	48.5		44.0
Viral load (≥ 500)^a^	32.1	22.6	19.4	< 0.001	26.0
HAART	73.4	76.1	76.7	< 0.001	75.0
*Comorbid mental and physical health conditions*					
Smoking status					
Never	24.5	30.9	29.5	< 0.001	27.1
Past	10.3	17.1	16.9		13.6
Current	65.3	51.9	53.6		59.3
Drug use	24.3	8.6	14.5	< 0.001	16.9
BMI^a^					
Underweight	3.7	1.5	2.5	< 0.001	3.0
Normal and overweight	76.7	79.7	79.5		78.2
Obese	19.6	18.8	18.0		18.8
Cancer (non-AIDS related)	6.4	7.0	12.6	< 0.001	9.1
Lung disease	17.5	17.6	19.9	< 0.001	18.5
Cardiovascular disease	12.2	11.8	15.1	< 0.001	13.4
Hypertension	49.2	38.4	40.3	< 0.001	44.5
Diabetes	14.3	15.8	12.2	< 0.001	13.5
Hepatitis B	10.2	13.2	8.1	< 0.001	9.5
Hepatitis C	28.4	14.7	26.0	< 0.001	22.3
Anxiety	11.6	18.9	18.8	< 0.001	15.3
Major depression	20.5	21.8	21.7	0.180	21.1
Psychosis (schizophrenia, schizoaffective, other)	13.2	11.8	13.6	0.014	12.6
PTSD	13.3	10.7	12.3	< 0.001	12.1
*AUDIT-C categories*					
AUDIT-C category					
Lower-risk (1–3)	74.2	76.9	78.2	< 0.001	76.1
Moderate-risk (4–7)	18.3	16.9	17.1		17.7
High-risk (8–12)	7.5	6.3	4.8		6.2

^a^Missing data about disease severity makes N = 14,880, N for BMI = 17,188, N for smoking = 16,958

Mortality rates varied across racial ethnic groups (Table 2). Among all PLWH reporting past-year alcohol use and those reporting lower-risk use, black patients had the highest mortality rates relative to Hispanic and white patients (p = 0.01 and < 0.01 respectively). There were no significant differences in mortality rates across racial/ethnic groups among patients in the moderate or high-risk levels of alcohol use. As alcohol risk groups increased, mortality rates also increased overall and across all racial/ethnic groups.

**Table 2:** Mortality rates overall and by level of alcohol use and race for black, Hispanic, and white VA patients living with HIV (n = 17,239)

	Overall (n = 17, 239)		Black (n = 8518)		Hispanic (n = 1353)		White (n = 7368)		p value*
# of Deaths	2041		1042		128		871		*0.014*

	Mortality rate^a^	95% CI	Mortality rate^a^	95% CI	Mortality rate^a^	95% CI	Mortality rate^a^	95% CI	p value*
Overall (n = 17,239)	2.7	(2.6, 2.9)	2.9	(2.7, 3.1)	2.2	(1.8, 2.6)	2.7	(2.5, 2.9)	*0.0111*
Lower-risk (n = 13,119)	2.4	(2.3, 2.6)	2.7	(2.5, 2.9)	1.8	(1.4, 2.2)	2.3	(2.1, 2.5)	*0.0008*
Moderate-risk (n = 3044)	3.3	(3.0, 3.7)	3.1	(2.7, 3.5)	2.9	(2.0, 4.2)	3.7	(3.2, 4.3)	0.1507
High risk (n = 1076)	5.0	(4.4, 5.7)	4.5	(3.7, 5.3)	5.0	(3.2, 8.0)	5.9	(4.8, 7.3)	0.1419

^a^Mortality rate is calculated per 100 person-years

*p value determined by Chi square test for difference in number of deaths and log-rank test for difference in mortality rate, italicized if significant

In all Cox Proportional Hazards models, the association between levels of alcohol use and mortality varied significantly across racial/ethnic groups (p value of overall interactions for Models 1–4 < 0.001). Associations between level of alcohol use and mortality risk within racial/ethnic groups differed based on covariate adjustment (Table 3). In unadjusted models (Model 1), and models adjusted for age only (Model 2), moderate- relative to lower-risk alcohol use was associated with significantly increased mortality risk for all patients across racial/ethnic groups. In models additionally adjusted for disease severity (Model 3), moderate- relative to lower-risk alcohol use was significantly associated with increased mortality risk only for whites and Hispanics. In models adjusted additionally for comorbidities (Model 4), moderate- relative to lower-risk alcohol use was associated with significantly increased mortality risk only for white patients (Table 3). For all racial/ethnic groups, across all models (Models 1–4), high-risk alcohol use was significantly associated with greater mortality risk than lower-risk alcohol use. The magnitude of the association appeared to differ such that Hispanic and white PLWH had higher mortality risk than black PLWH at high-risk levels of alcohol use relative to lower-risk levels, although the confidence intervals overlapped in many cases.

**Table 3:** Hazard ratios of mortality comparing moderate- and high-risk relative to lower-risk alcohol use among black, Hispanic, and white VA patients living with HIV: overall and by race/ethnicity

Alcohol use categories	Iterative modeling strategy	Overall		Black		Hispanic		White	
		(n = 17,239)		(n = 8518)		(n = 1354)		(n = 7376)	
		HR	95% CI	HR	95% CI	HR	95% CI	HR	95% CI
Lower-risk		Ref							
Moderate-risk	Model 1^a^	*1.37*	*(1.23, 1.53)*	*1.17*	*(1.00, 1.36)*	*1.63*	*(1.06, 2.49)*	*1.60*	*(1.37, 1.88)*
	Model 2^b^	*1.39*	*(1.25, 1.54)*	*1.20*	*(1.03, 1.40)*	*1.64*	*(1.07, 2.51)*	*1.59*	*(1.35, 1.86)*
	Model 3^c^	*1.34*	*(1.21, 1.50)*	1.15	(0.99, 1.34)	*1.56*	*(1.02, 2.39)*	*1.56*	*(1.33, 1.84)*
	Model 4^d^	*1.26*	*(1.13, 1.40)*	1.08	(0.92, 1.26)	1.36	(0.89, 2.08)	*1.50*	*(1.27, 1.76)*
High-risk	Model 1^a^	*2.04*	*(1.77, 2.35)*	*1.68*	*(1.39, 2.04)*	*2.80*	*(1.68, 4.66)*	*2.55*	*(2.03, 3.20)*
	Model 2^b^	*2.11*	*(1.83, 2.43)*	*1.66*	*(1.37, 2.02)*	*2.65*	*(1.59, 4.41)*	*2.85*	*(2.27, 3.58)*
	Model 3^c^	*1.92*	*(1.66, 2.21)*	*1.52*	*(1.25, 1.85)*	*2.49*	*(1.49, 4.15)*	*2.61*	*(2.08, 3.29)*
	Model 4^d^	*1.62*	*(1.40, 1.88)*	*1.35*	*(1.11, 1.64)*	*2.14*	*(1.28, 3.58)*	*2.01*	*(1.59, 2.55)*

Interactions between alcohol use categories and racial/ethnic groups were all significant (p < 0.001)

Italicsed results are significant at p value < 0.05

^a^Model 1 is unadjusted

^b^Model 2 is adjusted for age

^c^Model 3 is adjusted for age and CD4 count

^d^Model 4 is adjusted for age, disease severity (CD4 count), receipt of HAART, and comorbidities (non-AIDS related Cancers (bladder, breast, buccal cavity and pharynx, digestive system, Hodgkin’s lymphoma, ill-defined cancer, kidney, leukemia, male genital system, melanomas, non-epithelial skin cancer, penile, prostate, stomach, testicular, ureter, urinary system), lung disease (COPD, asthma, or other lung disease), cardiovascular disease (congestive heart failure, MI/CAD, peripheral vascular disease, ischemic stroke, stroke), hypertension, diabetes, hepatitis B, hepatitis C, anxiety, psychosis (schizophrenia, schizoaffective disorder, other psychosis), PSTD, BMI, drug use, and lifetime tobacco use

To examine magnitude of associations across racial/ethnic differences, the effect of race/ethnicity on mortality risk within each AUDIT-C category was considered in secondary analysis (Table 4). Black patients had a significantly higher mortality risk compared to white and Hispanic patients at lower-risk levels of alcohol use in unadjusted models (Model 1), when adjusted for age only (Model 2), and when adjusted for age and HIV disease severity (Model 3). When comparing mortality risk across racial/ethnic groups at lower-risk levels of alcohol use in models adjusted for age, disease severity, and comorbidities (Model 4), black patients had significantly higher mortality risk than Hispanics, while Hispanics had lower mortality risk than white PLWH. Among PLWH reporting moderate-risk alcohol use, the only significant difference across racial ethnic groups was in fully adjusted models, such that both black and Hispanic patients had lower mortality risk than white patients. Among PLWH reporting high-risk alcohol use, there were no significant differences between Hispanic relative to white, nor among black relative to Hispanic patients (Models 1–4). However, in all models (Models 1–4) among patients reporting high-risk alcohol use, black patients had significantly lower mortality risk than white patients.

**Table 4:** Hazard ratios comparing mortality between racial/ethnic groups within each AUDIT-C category

Comparison by race/ethnicity	Lower-risk		Moderate-risk		High-risk	
	HR	95% CI	HR	95% CI	HR	95% CI
Black relative to white						
Model 1^a^	*1.15*	*(1.03, 1.28)*	0.84	(0.69, 1.01)	*0.76*	*(0.57, 1.00)*
Model 2^b^	*1.25*	*(1.12, 1.39)*	0.94	(0.77, 1.14)	*0.73*	*(0.55, 0.96)*
Model 3^c^	*1.17*	*(1.05, 1.31)*	0.86	(0.71, 1.05)	*0.68*	*(0.52, 0.90)*
Model 4^d^	0.99	(0.88, 1.11)	*0.71*	*(0.58, 0.87)*	*0.66*	*(0.50, 0.87)*
Hispanic relative to white						
Model 1^a^	*0.78*	*(0.61, 0.98)*	0.79	(0.53, 1.16)	0.85	(0.51, 1.41)
Model 2^b^	0.83	(0.66, 1.05)	0.86	(0.58, 1.27)	0.77	(0.46, 1.29)
Model 3^c^	0.81	(0.64, 1.02)	0.81	(0.55, 1.19)	0.77	(0.46, 1.28)
Model 4^d^	*0.75*	*(0.59, 0.94)*	0.68	(0.46, 1.00)	0.80	(0.48, 1.33)
Black relative to Hispanic						
Model 1^a^	*1.48*	*(1.17, 1.86)*	1.06	(0.72, 1.57)	0.89	(0.54, 1.46)
Model 2^b^	*1.50*	*(1.19, 1.89)*	1.09	(0.74, 1.61)	0.94	(0.57, 1.54)
Model 3^c^	*1.45*	*(1.15, 1.82)*	1.07	(0.73, 1.58)	0.89	(0.54, 1.46)
Model 4^d^	*1.32*	*(1.05, 1.66)*	1.05	(0.71, 1.55)	0.83	(0.50, 1.37)

^a^Model 1 is unadjusted

^b^Model 2 is adjusted for age

^c^Model 3 is adjusted for age and CD4 count

^d^Model 4 is adjusted for age, disease severity (CD4 count), receipt of HAART, and comorbidities (non-AIDS related Cancers (bladder, breast, buccal cavity and pharynx, digestive system, Hodgkin’s lymphoma, ill-defined cancer, kidney, leukemia, male genital system, melanomas, non-epithelial skin cancer, penile, prostate, stomach, testicular, ureter, urinary system), lung disease (COPD, asthma, or other lung disease), cardiovascular disease (congestive heart failure, MI/CAD, peripheral vascular disease, ischemic stroke, stroke), hypertension, diabetes, hepatitis B, hepatitis C, anxiety, psychosis (schizophrenia, schizoaffective disorder, other psychosis), PSTD, BMI, drug use, and lifetime tobacco use

Italicsed results are significant at p value < 0.05

## Discussion

In this national sample of male PLWH who receive VA care, associations between alcohol use and mortality varied across racial/ethnic groups. For all racial/ethnic groups, high-risk relative to lower-risk alcohol use was associated with increased risk of mortality. Moderate relative to lower-risk alcohol use was associated with statistically significant increased mortality risk for all racial ethnic groups in unadjusted models, but only among white patients in fully adjusted models. For black and Hispanic PLWH, point estimates suggested increased risk associated with moderate levels of alcohol use, but no statistically significant associations were observed in fully adjusted models.

These findings expand on current understanding of alcohol-related risk among PLWH. While previous studies among PLWH identified greater risk of mortality for patients with both moderate- and high-risk alcohol use relative to those with lower-risk alcohol use [4, 28], findings from the present study suggest that these associations vary across racial/ethnic groups. Differences in the association between alcohol use and mortality may be driven by racial/ethnic differences in patients reporting high-risk or moderate-risk alcohol use, by differences in mortality risk among those reporting lower-risk alcohol use, which were used as a reference group in analyses comparing levels of alcohol use, or by residual confounding. While previous research suggested that racial/ethnic minorities may be at increased risk of mortality associated with alcohol use, our findings suggest a more complicated picture.

In this study, Hispanic PLWH had increased mortality among patients reporting high-risk, but not moderate-risk alcohol use, relative to those reporting lower-risk alcohol use, but decreased mortality relative to black patients reporting lower-risk levels of use in all models and marginally decreased mortality risk relative to white patients in unadjusted and fully adjusted models. There were fewer Hispanic patients than other racial/ethnic groups in this sample, and the model may have been insufficiently powered to detect differences among Hispanic patients. Further, previous research on alcohol use among Hispanics identified differences in alcohol-related outcomes based on acculturation to the United States, with higher levels of acculturation being associated with poorer alcohol-related outcomes [45, 46]. In this secondary data analysis, we did not have access to measures regarding contextual or cultural factors, thus, there may be unmeasured protective factors in this group that build resiliency against the ill effects of alcohol use.

Among black PLWH reporting any alcohol use, there was no significant difference in mortality risk among patients with moderate-risk relative to lower-risk alcohol use in fully adjusted models, although there was significant increased risk of mortality for high- relative to lower-risk alcohol use. These findings are consistent with previous research in general (non-HIV specific) outpatients [17]. Our comparisons of racial/ethnic groups within alcohol use levels may help explain these findings. Specifically, we found that black patients had increased mortality relative to white patients among PLWH reporting lower risk alcohol use, although this was attenuated by adjustment for comorbid conditions. Additionally, black patients had decreased mortality risk relative to white patients at higher-risk levels of alcohol use.

Reasons for the lack of increased risk of mortality among patients reporting moderate- and high-risk levels of alcohol use relative to those reporting lower-risk alcohol use among black PLWH are unknown, but may be due to contextual factors relating to alcohol use and competing health conditions. For example, previous studies have suggested that greater prevalence of adverse alcohol-related consequences among black relative to white persons [10, 16] relates to greater exposure to racism, and community-level contextual factors such as residence in low socioeconomic status areas leading to greater stress, with social norms promoting abstinence in black communities possibly leading to increased stigma for people who use alcohol, and surveillance by authorities leading to increased law enforcement involvement in black communities [10, 47–49]. However, there was no significant difference in alcohol-associated mortality risk comparing black to white patients at lower-levels of alcohol use when additionally adjusting for comorbidities (Model 4; Table 4), suggesting that competing health conditions may account for differences in mortality risk between black and white patients at lower-risk levels of alcohol use, and alcohol use may not be the strongest risk factor for death among black PLWH.

Competing health conditions may be more strongly associated with mortality, and may be bi-directionally associated with alcohol use. Thus, adjusting for health factors that may be exacerbated by alcohol use may have over-adjusted findings and attenuated the association between alcohol use and mortality. Black patients have increased risk of both prevalence of adverse health conditions and poor outcomes associated with these conditions relative to white patients, in part owing to substantial stigma and discrimination experienced by black persons due to racism [50]. For instance, in this sample, black patients had the highest proportion of hypertension, Hepatitis C, current tobacco use, and other drug use. These conditions may be more prevalent among black patients [51] or more strongly associated with poor outcomes among black patients [52, 53] due to contextual factors including racism [50, 54], and all are strongly predictive of mortality among PLWH [55–58]. Additionally, these conditions are bi-directionally associated with alcohol use [59, 60], and adjusting for these possible mediating factors may have over-adjusted the model. These measures, included only in Model 4 and disproportionately distributed among black patients, may have inadvertently adjusted away some pathways (e.g., racism) via which alcohol use would be associated with mortality for black PLWH.

Finally, it is possible that differential receipt of specialty addictions treatment across racial/ethnic groups could help explain findings of the present study. Specifically, black patients are more likely than white to receive specialty addictions treatment in the VA system [61, 62]. Therefore, it is possible that higher treatment receipt among black patients may account for lack of association between moderate-risk levels of alcohol use and mortality among black patients, and differences in mortality risk at high-risk levels of alcohol use between black and white patients. Future research is needed to investigate this issue.

The overall patterns observed in the data across racial/ethnic groups replicate findings from a similar study regarding associations between alcohol use and mortality among non-HIV-specific VA patients [17]. While previous studies show that non-white patients have worse consequences of alcohol use at similar levels to white patients [10, 16], there may be aspects of care at the VA that make this populations unique. Eligibility for VA services reduces disparities in access to care, which may reduce racial/ethnic disparities overall [63]. Additionally, there may be increased mortality risk for white PLWH at moderate risk levels of alcohol use, but not for racial/ethnic minorities, perhaps due to risk factors that make PLWH unique. Future research is needed to better understand and address contributing factors mediating the strength of associations between alcohol use and mortality risk.

Findings confirm risks of alcohol use at high-risk levels for all racial/ethnic groups and affirm recommendations for clinical interventions. Specifically, brief interventions are recommended for all primary care patients who drink above recommended limits, and specialty addictions treatment or alcohol use disorder medications are recommended for patients with more severe unhealthy alcohol use [64]. PLWH are at increased risk of under-receipt of alcohol-related treatment, relative to patients without HIV [65, 66], and findings of the present study further underscore the need to address unhealthy alcohol use among PLWH. Increased access to alcohol-related care is needed, and should be offered across treatment settings, including both in primary care and HIV treatment settings [67–70].

There are several limitations to this study. This study used secondary clinical data and was observational. Thus, residual confounding (e.g., by socioeconomic status, sexual orientation, gender identity, or rural status, which were unmeasured or not included in this dataset) may be present. Socioeconomic status, in particular, is a known risk factor for poor outcomes among PLWH for which we did not have a measure [71], and adjustment for it decreased the association between alcohol use and mortality in a previous study [17]. Similarly, the study design did not allow identification of entry into VA care, which may be patterned by race and influence mortality and thus reflect an additional residual confounder. While the AUDIT-C has been validated across racial/ethnic groups [32], black patients may be more likely to under-report alcohol use due to stigma [72]. A previous study in VACS additionally showed that among patients reporting past-year abstinence from alcohol use, those with HCV had increased likelihood of biomarker-detected alcohol use [28], and black patients are overrepresented among VACS patients with HCV relative to those without HCV [73]. Black patients may also be more likely to overestimate the size of a standard drink [74], which may have misclassified alcohol use level among black patients in this study. Additionally, the AUDIT-C, while a validated screen for unhealthy alcohol use [23–25], is not an in-depth structured assessment of alcohol use. In this cohort, AUDIT-C is asked and documented by a clinician, and may be limited based on method of screening administration and results documentation [75], as well as by patient recall or social desirability bias [76]. Further, though alcohol use can vary over time within persons, alcohol use was also not considered to be a time-varying factor in this analysis. However, previous research assessing repeated alcohol screening scores [77] and research using AUDIT-C trajectory models indicates that the vast majority of patients remain in stable AUDIT-C categories over time [78]. Additionally, missing data, which was accounted for in this study using multiple imputation, may not be missing at random. There are also limitations to the external validity of this study. This study is only among men receiving care at the VA, and may not be generalizable to women veterans living with HIV and/or non-veterans. Additionally, PLWH in this study are already linked with care, and results will not be generalizable to PLWH not linked with care. Finally, alcohol use data is only available from 2008, when the AUDIT-C screening data became widely available in the VA. Future studies are needed examining these issues among women, non-veterans, and people living with HIV not linked to healthcare.

Future research is also needed to determine which factors mediate the association between alcohol use and mortality to understand variation in the association across racial/ethnic groups. Better understanding these mechanisms will be integral for designing policies and interventions to prevent mortality associated with alcohol use among all PLWH. Our findings are hypothesis-generating regarding potential mechanisms. For instance, our findings suggest the possibility that there were competing risks for mortality among more vulnerable racial/ethnic groups, as comorbid conditions and greater HIV disease severity were over-represented among black persons in this population. Similarly, white patients may have been more susceptible to the effects of alcohol use on ART. They were more likely to be on ART, which may have increased the influence of heavy alcohol use on risk of death (by affected adherence to ART) relative to black persons (who were less likely to receive ART). The influence of other potential moderating factors, such as receipt of alcohol-related care and rural status, should also be examined.

 Despite limitations and need for further research, this study—conducted in a national sample of racially/ethnically diverse PLWH receiving care from the largest single provider of HIV care in the United States—is the first to our knowledge to evaluate the influence of alcohol use on mortality across racial/ethnic minority groups of PLWH. Findings from this study suggest that, similar to studies among general outpatients [17], associations between alcohol use and mortality vary across racial/ethnic groups. While associations vary in magnitude across racial/ethnic groups and may be dependent on other patient level factors within racial/ethnic groups, overall patterns were similar across groups. This highlights the need to increase access to alcohol-related care to address high-risk alcohol use across all racial/ethnic groups to decrease mortality risk among PLWH, while considering potential that competing health conditions may account for increased mortality risk among racial/ethnic minority PLWH at lower-risk levels of alcohol use.
